# Impact of preoperative lumbosacral takeoff flexibility on postoperative correction following spinal fusion for adolescent idiopathic scoliosis: a new consideration for selective thoracic fusion

**DOI:** 10.1007/s43390-025-01063-6

**Published:** 2025-03-13

**Authors:** Richard E. Campbell, Theodore Rudic, Alexander Hafey, Elizabeth Driskill, Peter O. Newton, Keith R. Bachmann, Harms Research Study Group, Keith R. Bachmann, A Noelle Larson, Aaron Buckland, Ahmet Alanay, Amer Samdani, Amit Jain, Baron Lonner, Benjamin Roye, Burt Yaszay, Caglar Yilgor, Dan Hoernschmeyer, Daniel Hedequist, Daniel Sucato, David Clements, Firoz Miyanji, Harry Shufflebarger, Jack Flynn, Jean Marc Mac Thiong, Josh Murphy, Joshua Pahys, Kevin Neal, Laurel Blakemore, Lawrence Haber, Lawrence Lenke, Mark Abel, Mark Erickson, Michael Glotzbecker, Michael Kelly, Michael Vitale, Michelle Marks, Munish Gupta, Nicholas Fletcher, Patrick Cahill, Paul Sponseller, Peter Gabos, Peter Newton, Peter Sturm, Randal Betz, Robert H Cho, Stefan Parent, Stephen George, Steven Hwang, Suken Shah, Sumeet Garg, Tom Errico, Vidyadhar Upasani

**Affiliations:** 1 Department of Orthopedic Surgery, University of Virginia Health System, PO Box 800159, Charlottesville, VA 22908 USA; 2 Department of Orthopaedic Surgery, Cleveland Clinic, Cleveland, OH USA; 3 Department of Orthopaedic Surgery, Medical University of South Carolina, Charleston, SC USA; 4 Division of Orthopedics and Scoliosis, Rady Children’S Hospital, San Diego, CA USA

**Keywords:** Scoliosis, LSTOA, Bending radiographs, Selective fusion

## Abstract

**Purpose:**

Nonselective fusion for adolescent idiopathic scoliosis results in greater correction of the Lumbosacral Takeoff Angle (LSTOA); however, there are patients selectively fused that still have considerable change in their LSTOA. We sought to identify the relationship between preoperative LSTOA flexibility and postoperative correction of the LSTOA.

**Methods:**

This was a retrospective analysis of Lenke 1–6, lumbar B and C modifier patients in the Harms Study Group with 2-year follow-up. Only patients with a lumbar Cobb angle ≥ 38 and ≤ 56 were included. The cases were divided into selective (SF: 177) and nonselective fusions (NSF: 324). Multivariate regression analysis was used to identify independent preoperative factors associated with postoperative LSTOA, and postoperative LSTOA correction in the NSF and SF groups.

**Results:**

The mean postoperative LSTOA correction was 6.1 ± 3.8, with 75 (15%) patients experiencing postoperative worsening of their LSTOA. Among other variables, larger LSTOA (p < 0.001) and smaller bending LSTOA correction (p < 0.001) were predictors of larger postoperative LSTOAs in both groups. Among other variables, larger LSTOA (p < 0.001), and larger bending LSTOA correction (p < 0.01) were predictors of greater LSOTA correction in both groups. Satisfactory LSTOA correction in the selective fusion group was associated with larger preoperative LSTOA (p < 0.001), larger bending LSTOA correction (p < 0.001), larger lumbar Cobb angle bending correction (p: 0.034), and smaller lumbar apex to LIV distance (p: 0.003).

**Conclusions:**

Preoperative static and bending LSTOA measurements may help surgeons decide between selective and non-selective fusion in patients with AIS.

**Level of evidence:** 3

## Introduction

Spinal fusion for adolescent idiopathic scoliosis (AIS) seeks to balance correction of deformity with maintained mobility. The recent surge of interest in non-fusion methods highlights the concern for the adverse effects associated with extensive fusions [1–10]. Selective thoracic fusion preserves mobility by sparing lumbar fusion levels; however, this comes at the potential expense of residual lumbar deformity. The Lenke classification introduced criteria intended to provide a standardized method for determining which patients are appropriate candidates for selective fusion on the basis of the relative magnitude of the lumbar and thoracic curves, their flexibility in bending, sagittal plane position, and the translation of the apical vertebra [11]. While this system has become the standard for the classification of AIS, retrospective studies show that surgeons frequently deviate from the guidelines when choosing between selective and nonselective fusion [12]. In addition, several studies have shown that there is a large overlap in the magnitude of the preoperative lumbar Cobb angles in patients who undergo selective and nonselective fusion [12–15]. These findings indicate that there is a lack of consensus among surgeons regarding which curves can be managed with selective fusion.

We have taken an interest in the role of the Lumbo-Sacral Takeoff Angle (LSTOA) as a tool independent of the Lenke classification to help guide selection of fusion levels. The LSTOA is defined as the angle between the center-sacral vertical line and a best-fit line drawn through the centroid of S1, L5, and L4 [16]. In previous studies, we showed that the preoperative LSTOA can be used to predict the postoperative lumbar Cobb angle after selective thoracic fusion in patients with Lenke 1B, 1C, 3B, and 3C curves [13, 16]. We also noted that the LSTOA change is maximized with distal fusion level approaching the sacrum; however, there were still patients with a last instrumented vertebra (LIV) cranial to the lumbar apex with appreciable change in their LSTOA. Identification of patient characteristics indicative of large LSTOA correction despite selective fusion can aid surgeons when deciding between selective or nonselective fusions.

**Fig. 1:**
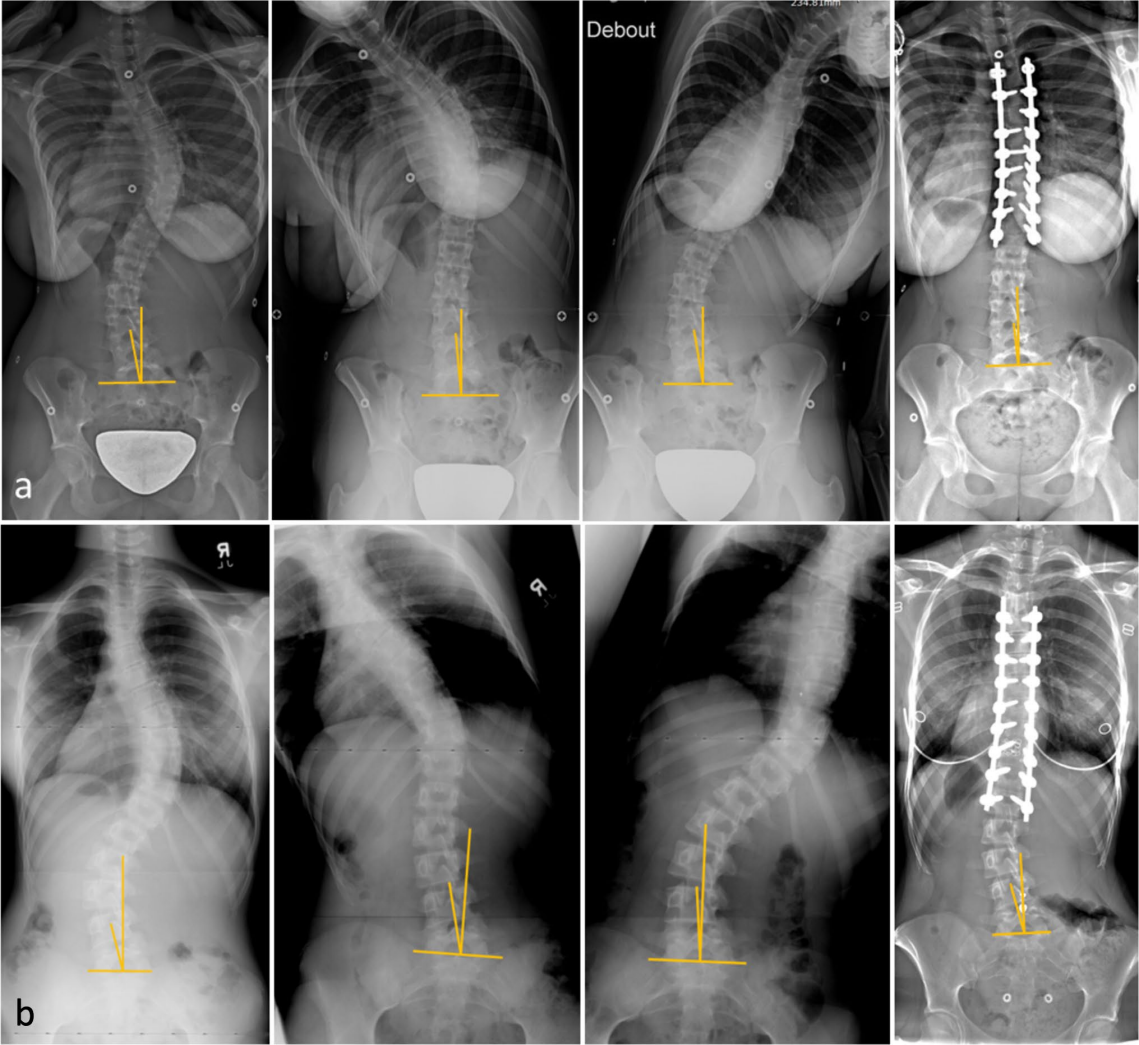
**a** Preoperative and postoperative PA radiographs of ‘flexible’ LSTOA, with an initial LSTOA of 8.5 that measured 2.2 with ipsilateral bending and 3.2 postoperatively. **b** Preoperative and postoperative PA radiographs of ‘ridged’ LSTOA, with an initial LSTOA of 15.5 that measured 15.8 with ipsilateral bending and 15.6 postoperatively

Similar to the thoracic and lumbar curve, the LSTOA can change with lateral bending to varying degrees (Fig. 1). While the relationship between preoperative bending thoracic and lumbar Cobb angles, and postoperative outcomes have been studied, the effect of preoperative LSTOA flexibility is still unknown. The purpose of this study was to identify the relationship between preoperative bending LSTOA and postoperative LSTOA correction, as well as identify predictors of change of LSTOA in a larger more inclusive group of patients undergoing posterior spinal fusion for AIS. We hypothesized that preoperative bending LSTOA flexibility would correlate with postoperative change in LSTOA.

## Methods

**Fig. 2:**
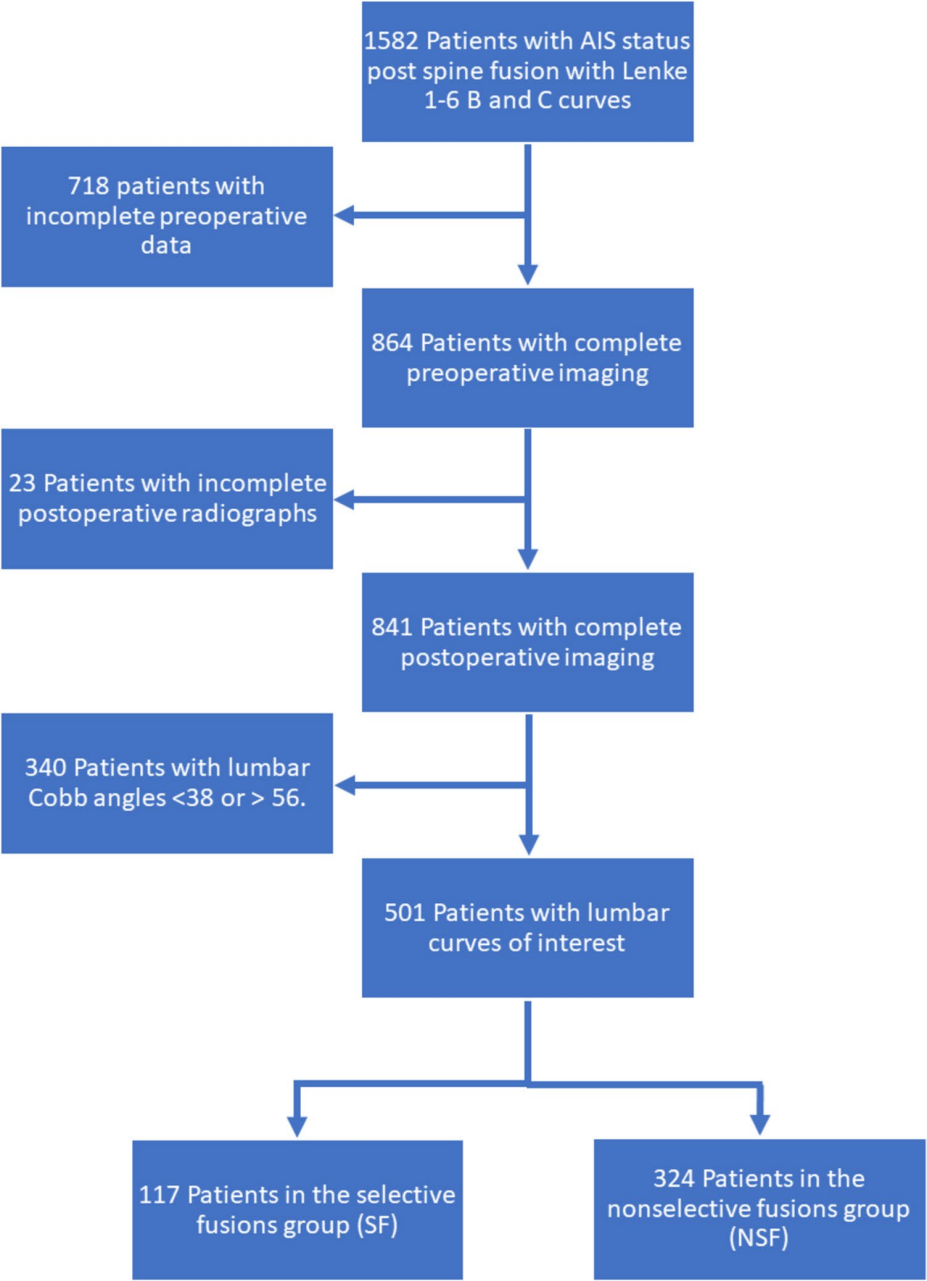
Flow diagram depicting patient exclusion and categorization within this study

This was a retrospective analysis of patients from a prospectively collected multi-center study group database with minimum 2 year follow-up. The database was queried to identify patients with Lenke 1–6 curves who underwent posterior spinal fusion for AIS. Patients with Lenke lumbar A modifier curves were excluded, as the preoperative lumbar deformity is small in these patients. Patients without preoperative and 2 year postoperative LSTOA measurements, as well as preoperative bending LSTOA measurements were also excluded. Patient information including demographics, clinical data, radiographic data, and Scoliosis Research Society-22R (SRS) scores, was obtained from the database. Consent for inclusion in this database and institutional review board approval was obtained prior to data collection. Patients were divided into selective (SF) and nonselective fusions (NSF), with selective fusions including patients with an LIV at or cranial to the lumbar apex. This definition was utilized, because it is consistent with prior studies evaluating the LSTOA in nonselective and selective fusions, in which the change in LSTOA was found to be related to the distance between the LIV and lumbar apex.[13, 16]. To focus on patients that may be indicated for both selective or nonselective fusion, patients with a preoperative lumbar Cobb angle > 56 or < 38 were also excluded, as 95% of patients with selective fusions had a lumbar Cobb angle ≤ 56°, and 95% of patients with nonselective fusions had a lumbar Cobb angle ≥ 38° (Fig. 2).

To identify patients within the SF group with LSTOA correction similar to the NSF group, descriptive statistics of postoperative LSOTA correction in the NSF group were obtained. A cutoff value for satisfactory postoperative LSTOA correction in the SF group was set to the 25th percentile of the NSF group. This threshold was arbitrary in nature but created a group of patients with selective fusions with similar postoperative outcomes compared to the NSF group.

### LSTOA measurements

**Fig. 3:**
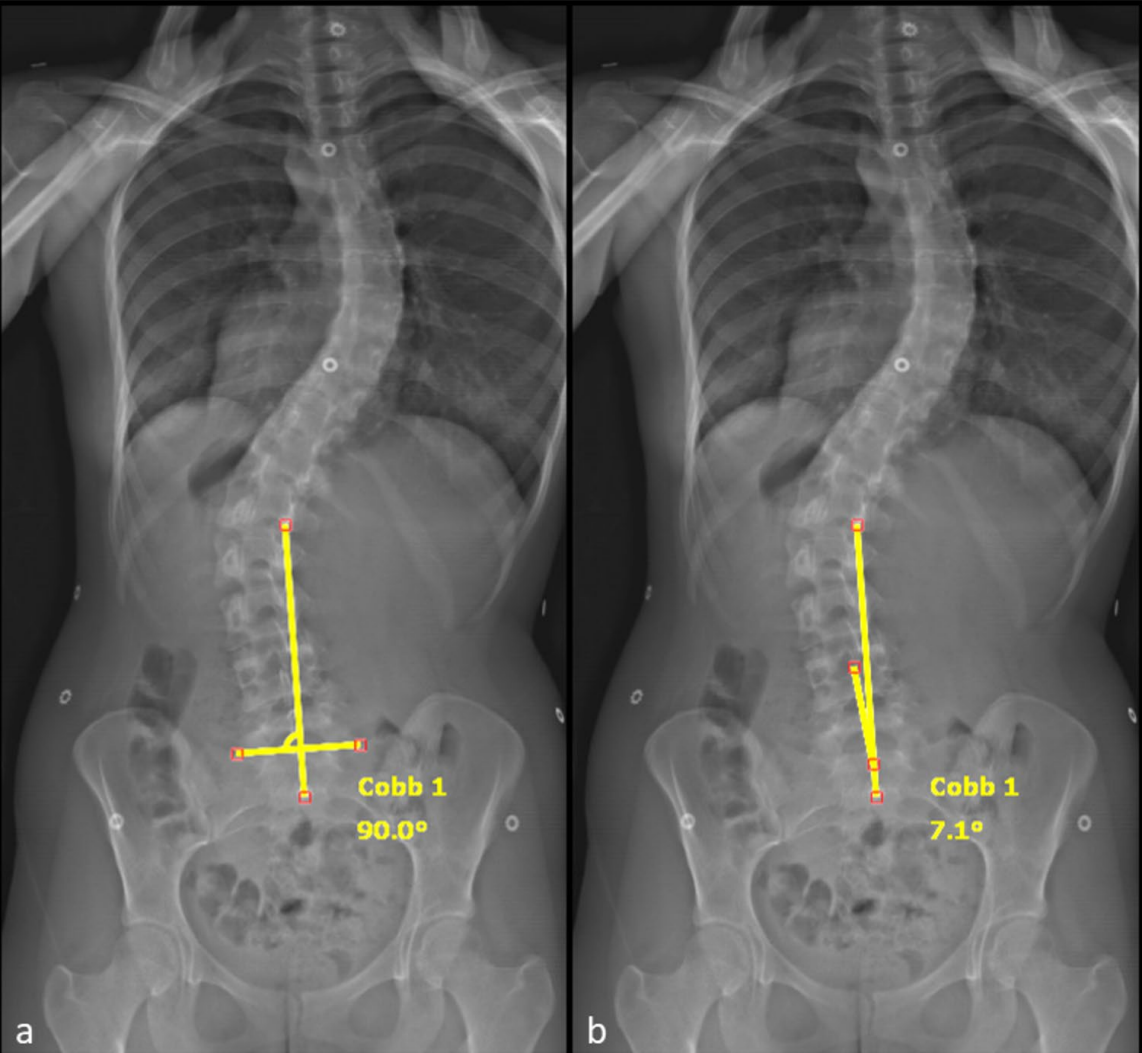
LSTOA measurement demonstrating a modified CSVL to account for sacral obliquity (**a**) and then measuring the LSTOA with a best fit line through the centroid of L4, L5, and S1 (**b**)

**Fig. 4:**
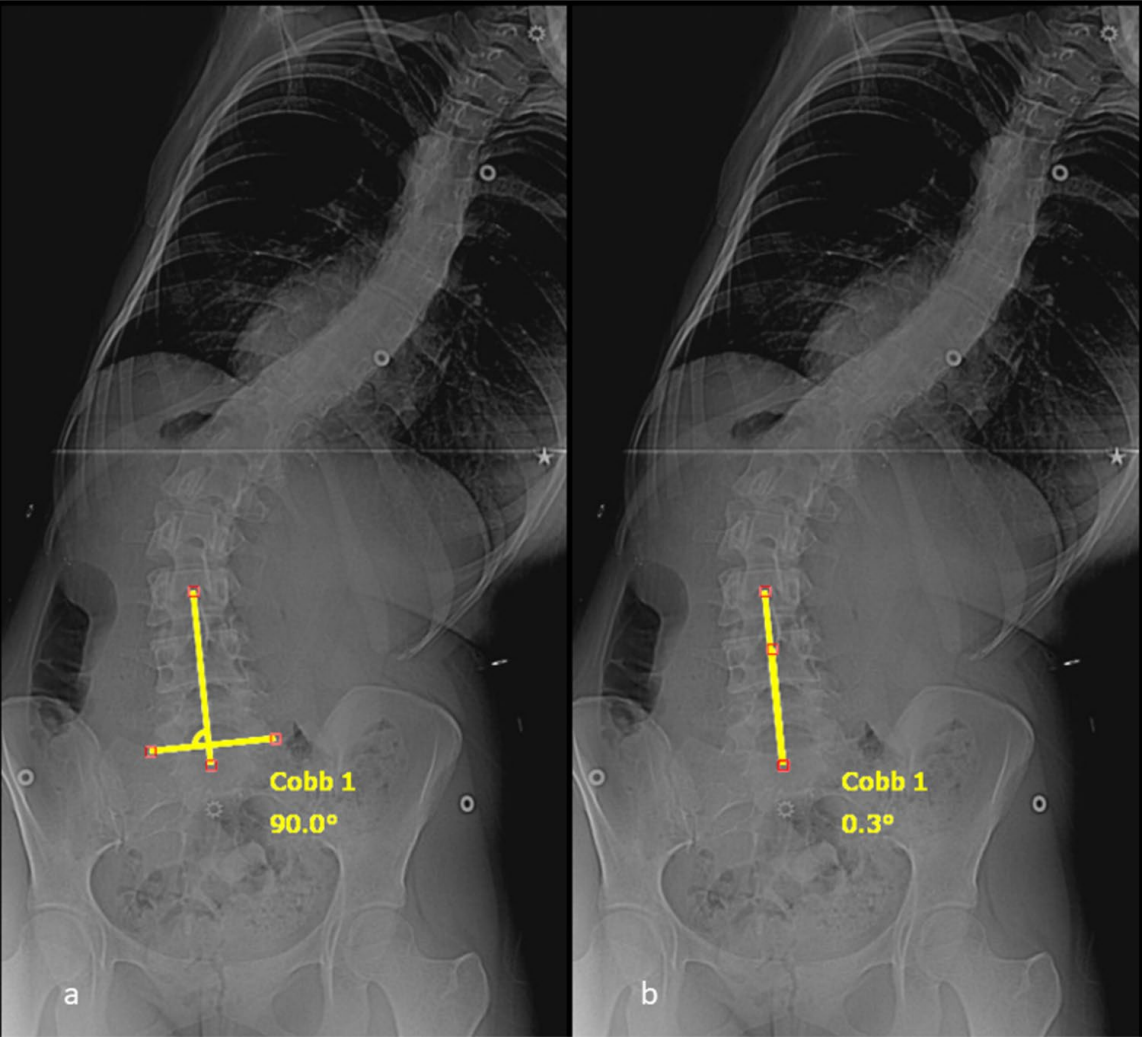
Bending LSTOA measurement for the patient in Fig. 2 demonstrating modified CSVL to account for sacral obliquity (**a**) and then measuring the LSTOA with a best fit line through the centroid of L4, L5, and S1 (**b**)

A modification of the LSTOA measurement, as described by Abel et al. [13] was made to allow measurement of a bending LSTOA. A line was drawn between the sacral ala and then a right angle created to this line. This vertical line would serve as the CSVL standardized to the pelvic obliquity of the patient. This technique was also used for the upright measurements of LSTOA. A second line was drawn as the best fit between the centrum of L4, L5, and S1 vertebrae, as demonstrated in Fig. 3, with an example of measuring the LSTOA on a bending X-ray in Fig. 4. Initially 20 patients randomly selected were measured by 3 different observers (A.H., T.R., and K.B.) to ensure adequate reliability. Cronbach’s alpha was 0.93, showing adequate reliability between raters and the remaining images were measured by A.H. and T.R. The standard error of measurement for LSOTA measurements was 1.41°. Bending LSTOA and lumbar correction was defined as the preoperative static angle minus the preoperative bending angle. Ipsilateral and contralateral LSTOA correction was defined as bending towards or away from the LSTOA side, respectively.

### Statistical analysis

The primary outcome was 2 year postoperative LSTOA correction, defined as the reduction in the magnitude (absolute value) from preoperative to postoperative. Secondary outcomes include 2 year postoperative LSTOA and the postoperative percentage decrease of the LSTOA. Continuous results are reported as mean ± standard deviation. *T* tests and Mann–Whitney *U* tests to analyze parametric and nonparametric data between groups. Pearson and Spearman’s rho correlations were calculated to determine the relationship between lumbar Cobb angles and LSTOAs. Pearson correlations were also calculated for parametric variables to determine inclusion in the linear regression models. We used multivariate linear regressions to identify independent predictors of postoperative LSTOA and LSTOA correction. Multivariate logistic regression was used to identify independent predictors of satisfactory postoperative LSTOA correction within the SF group. Preoperative variables assessed in the multivariate analyses included: age at time of surgery, open triradiate cartilage, gender, lumbar apex to LIV vertebrae distance, main thoracic Cobb angle, thoracic bending Cobb correction, lumbar Cobb angle, lumbar bending correction, LSTOA, LSTOA direction, bending LSTOA correction (ipsilateral and contralateral), coronal balance, thoracic apical translation, lumbar apical translation, LIV angulation, T2–T12 kyphosis, T5–T12 kyphosis, T10–L2 kyphosis, and T12-sacrum lordosis. We used an alpha level of 0.05 to delineate statistical significance.

## Results

A total of 501 patients with preoperative and postoperative LSTOA measurements were included in the analysis, including 426 (85.0%) females (Table 1). The mean lumbar Cobb angle improved from 46.3 ± 5.2 preoperative to 19.1 ± 7.5 postoperatively. No patients had worsening of their lumbar Cobb angles postoperatively. Preoperative LSTOA was 10.3 ± 4.8, with 454 (90.6%) left sided LSTOAs. The mean postoperative LSTOA was 6.1 ± 3.8. The mean postoperative LSTOA correction was 4.1 ± 4.4, with 75 (15.0%) patients experiencing postoperative worsening of their LSTOA. There was a very weak correlation between the lumbar Cobb angles and static LSTOAs (*r*: 0.15, *p* < 0.001). There was also a very weak correlation between ipsilateral bending LSTOAs and bending lumbar Cobb angles (*r*: 0.18, *p* < 0.001), as well as lumbar Cobb bending correction and LSTOA bending correction (*r*: 0.196, *p* < 0.001). For reference, there was also a very weak correlation between thoracic and lumbar Cobb angles (*r*: 0.171, *p* < 0.001), a weak correlation between thoracic and lumbar bending Cobb angles (*r*: 0.257, *p* < 0.001), and a moderate correlation between thoracic Cobb bending correction and lumbar Cobb bending correction (*r*: 0.41, *p* < 0.001).

**Table 1:** Preoperative patient characteristics

Preoperative variable	Count (%) or mean ± SD			*p* value
	Total cohort (*n*: 501)	Nonselective fusion (*n*: 324)	Selective fusion (*n*: 177)	
Female gender	426 (85.0%)	277 (85.5%)	149 (84.2%)	0.694
Open triradiate cartilage	31 (6.2%)	13 (4.0)	18 (10.2)	0.006
Lenke classification				
1	118 (23.6%)	37 (11.4%)	81 (45.8%)	< 0.001
2	53 (10.6%)	10 (3.1%)	43 (24.3%)	
3	60 (12.0%)	27 (8.3%)	33 (18.6%)	
4	38 (7.6%)	21 (6.5%)	17 (9.6%)	
5	151 (30.1%)	149 (46.0%)	2 (1.1%)	
6	81 (16.2%)	80 (24.7%)	1 (0.6%)	
Age	14.7 ± 2.1	14.9 ± 2.1	14.2 ± 2.1	< 0.001
Thoracic Cobb	45.5 ± 16.7	38.5 ± 15.5	58.4 ± 9.4	< 0.001
Lumbar Cobb	46.3 ± 5.2	47.4 ± 5.0	44.3 ± 5.0	< 0.001
Coronal balance distance	2.2 ± 1.3	2.7 ± 1.3	1.4 ± 0.9	< 0.001
Lumbar apical translation	3.9 ± 1.7	4.7 ± 1.4	2.4 ± 0.9	< 0.001

*SD* standard deviation

### Selective and nonselective fusions cohorts

The SF group consisted of 177 patients, while the NSF consisted of 324 patients. Preoperative radiographic parameters differed between the groups as well (Table 1). The NSF group had larger preoperative LSTOAs (*p*: 0.041), smaller postoperative LSTOAs (*p* < 0.001) and greater postoperative LSTOA correction compared to the SF group (*p* < 0.001; Table 2).

**Table 2:** Preoperative and postoperative LSTOA measurements

Preoperative variable	Count (%) or mean ± SD			*p* value
	Total cohort (*n*: 501)	Nonselective fusion (*n*: 324)	Selective fusion (*n*: 177)	
Left LSTOA direction	454 (90.6%)	279 (86.1%)	175 (98.9%)	< 0.001
Preoperative LSTOA	10.3 ± 4.8	10.6 ± 5.0	9.7 ± 4.4	0.041
Preoperative ipsilateral bending LSTOA	10.5 ± 5.6	10.3 ± 5.8	10.7 ± 5.3	0.556
Preoperative contralateral bending LSTOA	8.8 ± 4.8	8.5 ± 4.6	9.4 ± 5.1	0.039
Preoperative ipsilateral bending LSTOA correction	− 0.2 ± 4.5	0.3 ± 4.6	− 1.0 ± 4.2	0.004
Preoperative contralateral bending LSTOA correction	1.4 ± 5.2	2.1 ± 5.1	0.2 ± 5.0	< 0.001
Postoperative LSTOA	6.1 ± 3.8	5.3 ± 3.6	7.6 ± 3.7	< 0.001
Preoperative to postoperative LSTOA correction	4.1 ± 4.4	5.3 ± 4.3	2.1 ± 3.9	< 0.001

*LSTOA* lumbosacral take off angle, *SD* standard deviation

Linear regression analysis was used to identify independent factors associated with postoperative LSTOA, and postoperative LSTOA correction in the NSF and SF groups (Table 3). Independent preoperative predictors of larger postoperative LSTOA in the NSF group included: older age (*p*: 0.003), larger LSTOA (*p* < 0.001), smaller ipsilateral bending LSTOA correction (*p* < 0.001). Independent preoperative predictors of greater postoperative LSTOA correction in the NSF group included: younger age (p: 0.002), larger LSTOA (*p* < 0.001), larger ipsilateral bending LSTOA correction (*p*: 0.004) and greater number of vertebrae between the lumbar apex and LIV (*p*: 0.024). All LIVs in the NSF were caudal to the lumbar apex.

**Table 3:** Independent predictors of postoperative LSTOA and LSTOA change

Preoperative variable	Nonselective fusion			Selective fusion		
	Standardized β	95% CI	*p* value	Standardized β	95% CI	*p* value
2-year postoperative LSTOA						
Age	0.14	0.05–0.24	0.003	−	−	−
LSTOA	0.60	0.50−0.70	< 0.001	0.61	0.48 − 0.73	< 0.001
Ipsilateral bending LSTOA Correction	− 0.17	− 0.26 to − 0.07	< 0.001	−0.28	− 0.41 to − 0.15	< 0.001
C7 – CSVL distance	−	−	−	−0.13	−0.25 − 0	0.043
2-year postoperative LSTOA correction						
Age	− 0.13	− 0.21 to − 0.04	0.002	−	−	−
LSTOA	0.66	0.57−0.74	< 0.001	0.53	0.41 − 0.65	< 0.001
Ipsilateral bending LSTOA Correction	0.13	0.04–0.21	0.004	0.27	0.15 − 0.39	< 0.001
Lumbar apex to LIV vertebrae distance	− 0.10	− 0.18 to − 0.01	0.024	−	−	−
Lumbar bending Cobb correction	−	−	−	0.125	0.01–0.24	0.034
LIV angulation	−	−	−	− 0.17	− 0.28 to − 0.05	.004

*LSTOA* lumbosacral takeoff angle, *CI* Confidence interval

Independent preoperative predictors of larger postoperative LSTOA in the SF group included: larger LSTOA (*p* < 0.001), smaller ipsilateral bending LSTOA correction (*p* < 0.001) and smaller C7–CSVL distance (*p*: 0.043). Independent preoperative predictors of greater postoperative LSTOA correction in the SF group included: larger lumbar Cobb angle bending correction (*p*: 0.034), larger LSTOA (*p* < 0.001), larger ipsilateral bending LSTOA correction (*p* < 0.001), and smaller LIV angulation (*p*: 0.004).

Further attention was focused on postoperative LSTOA correction in the selective fusion group. Patients were deemed to have satisfactory postoperative LSTOA correction if their postoperative change in LSTOA was > 2.7 based on the methodology previously stated. A total of 71 (40.1%) patients in the selective group had satisfactory LSTOA correction with a similar average postoperative decrease in LSTOA compared to the NSF group (Table 4). Multivariate logistic analysis identified the following independent predictors of satisfactory postop LSTOA correction: larger preoperative LSTOA (*p* < 0.001; OR: 1.3, 95% CI 1.2–1.5), larger ipsilateral bending LSTOA correction (*p* < 0.001, OR: 1.3, 95% CI 1.1–1.5), larger lumbar Cobb angle bending correction (*p*: 0.034; OR: 1.1, 95% CI 1.0–1.1), and smaller number of vertebrae between the lumbar apex and LIV (*p*: 0.003; OR: 0.4, 95% CI 0.2–0.7).

**Table 4:** Satisfactory selective fusion postoperative LSTOA correction

Postoperative variable	Satisfactory SF	NSF	*p* value
LSTOA			
Mean + SD	6.4 ± 3.5	5.3 ± 3.6	0.012
Median (IQR)	6.2 (3.7˗ 8.4)	4.5 (2.4 ˗ 7.8)	
LSTOA correction			
Mean + SD	5.6 ± 2.8	5.3 ± 4.3	0.610
Median (IQR)	4.8 (3.6 ˗ 6.5)	5.2 (2.8 ˗ 7.9)	

*SD* standard deviation, *IQR* interquartile range, *SF* selective fusion, nonselective fusion

## Discussion

The LSTOA is emerging as a useful preoperative and postoperative radiographic assessment for AIS. However, there is limited research identifying predictive factors for postoperative LSTOA correction. Furthermore, the relationship between the LSTOA and other radiographic parameters is not fully understood. These results provide insight into correction of the LSTOA and demonstrate the independence of the LSTOA as a predictive factor.

Previously, Abel et al. reported that more cranial LIV selection resulted in less correction in postoperative LSTOA [13]. Similarly, our results demonstrate a greater correction in LSTOA and smaller absolute postoperative LSTOA in the NSF group compared to the SF group. However, a significant percentage of patients in the selective group demonstrated LSTOA correction similar to patients in the NSF group. Identification of factors predictive of greater LSTOA correction can lead to better understanding of AIS correction, and in-turn better postoperative outcomes.

The LSTOA demonstrates characteristics similar to lumbar Cobb angles. Regardless of the fusion length, a larger preoperative LSTOA was associated with a larger postoperative LSTOA and greater postoperative LSTOA correction. It is intuitive that a larger preoperative LSTOA would result in a larger postoperative LSTOA and would have greater range for correction. Although the LSTOA shares similar characteristics as the lumbar Cobb, it is still independent of the lumbar curve. We identified a weak correlation between lumbar Cobb angles and LSTOAs, which was small when compared to the relationship between thoracic and lumbar Cobb angles. This is also demonstrated by the 15% of patients with worsening postoperative LSTOA despite all patients demonstrating improvement in their lumbar Cobb angles.

Our results also demonstrate that within selective fusions, greater LSTOA flexibility on pre-operative bending films was associated with greater LSTOA correction, similar to patients undergoing nonselective fusion. If the LSTOA is not flexible on bending X-rays, there is increased risk for suboptimal LSTOA correction if a selective fusion is performed, or vice versa. If the LSTOA is small or flexible then more of the Lumbar Cobb is arising from the lower portion of the thoracic curve and will have more spontaneous correction with thoracic fusion.

This understanding may help determine LIV in borderline surgical cases and identify ‘Lenke rule-breakers.’ There are scenarios in which the lumbar Cobb and bend films might indicate a nonselective fusion by Lenke criteria; however, the patient may have a small or flexible LSTOA, and would have a small postoperative LSTOA even after selective fusion. We encourage surgeons to examine the LSTOA as a separate pre-operative variable when considering whether to fuse the thoracic curve only or to include both the thoracic and lumbar curves in the fusion. For this reason, we chose not to analyze these results within the Lenke classification.

While this study sheds light onto the properties of the LSTOA, further research is needed. The clinical significance of a large postoperative LSTOA is currently unknown, making an acceptable postoperative LSTOA limit difficult to establish. Similarly, the postoperative differences in LSTOA between the groups were small when compared with the standard error of measurement, making their interpretation difficult. Furthermore, if a thoracic level is chosen as the lowest instrumented vertebra and the LSTOA does not improve with fusion, what happens to the LSTOA over time? Does the LSTOA remain stable after fusion or does it slowly increase leading to an increase in lumbar Cobb over time? We are pursuing these avenues of further study with this data.

Although informative, this study does have limitations. The inclusion of multiple surgical institutions and surgeons decreases internal validity and creates potential confounders. Specifically, supine bending films were obtained at multiple centers, by multiple technicians. This introduces potential confounders and bias, which weaken our internal validity and ability to test our hypothesis. The threshold for ‘satisfactory LSTOA correction’ within the selective fusion group was arbitrary, but served to create a SF subgroup with similar LSTOA correction as the NSF group. We also did not identify specific cutoffs for preoperative LSTOA or LSTOA flexibility. By analyzing the LSTOA measurements as continuous variables instead of creating artificial binary cutoffs, we aimed to prevent misinterpretation and misapplication of our results. Our results demonstrate an overall trend regarding the impact of LSTOA flexibility on postoperative outcomes that surgeons can incorporate into their decision making. Finally, only 2 year postoperative outcomes are included, making mid- and long-term conclusions difficult.

## Conclusion

Preoperative static and bending LSTOA is an independent radiographic measure that may play a role in helping surgeons decide between selective and non-selective fusion in patients with AIS.

## Data Availability

Data is part of a prospective study group but selective portions of the data may be made available upon request to the corresponding author.
